# Sentinel-2 Data for Precision Agriculture?—A UAV-Based Assessment

**DOI:** 10.3390/s21082861

**Published:** 2021-04-19

**Authors:** Josephine Bukowiecki, Till Rose, Henning Kage

**Affiliations:** Institute of Crop Science and Plant Breeding, Christian-Albrechts-University, 24118 Kiel, Germany; rose@pflanzenbau.uni-kiel.de (T.R.); kage@pflanzenbau.uni-kiel.de (H.K.)

**Keywords:** Sentinel-2, UAV, GAI, winter wheat, precision agriculture

## Abstract

An approach of exploiting and assessing the potential of Sentinel-2 data in the context of precision agriculture by using data from an unmanned aerial vehicle (UAV) is presented based on a four-year dataset. An established model for the estimation of the green area index (GAI) of winter wheat from a UAV-based multispectral camera was used to calibrate the Sentinel-2 data. Large independent datasets were used for evaluation purposes. Furthermore, the potential of the satellite-based GAI-predictions for crop monitoring and yield prediction was tested. Therefore, the total absorbed photosynthetic radiation between spring and harvest was calculated with satellite and UAV data and correlated with the final grain yield. Yield maps at the same resolution were generated by combining yield data on a plot level with a UAV-based crop coverage map. The best tested model for satellite-based GAI-prediction was obtained by combining the near-, infrared- and Red Edge-waveband in a simple ratio (R^2^ = 0.82, mean absolute error = 0.52 m^2^/m^2^). Yet, the Sentinel-2 data seem to depict average GAI-developments through the seasons, rather than to map site-specific variations at single acquisition dates. The results show that the lower information content of the satellite-based crop monitoring might be mainly traced back to its coarser Red Edge-band. Additionally, date-specific effects within the Sentinel-2 data were detected. Due to cloud coverage, the temporal resolution was found to be unsatisfactory as well. These results emphasize the need for further research on the applicability of the Sentinel-2 data and a cautious use in the context of precision agriculture.

## 1. Introduction

In the context of sustainable intensification of food production systems (e.g., fertilization, irrigation, pesticide application), the estimation of spatially and temporally varying crop productivity as early and precise as possible is of major concern [1]. Herein, the availability of reliable and affordable data is a key issue, whereby the required time intervals and the spatial resolution differ with regard to the main objective and the considered crop. In the last decades, ground-based sensors were the most widely used option to obtain such data for precision farming [2]. Their main disadvantage is a limited area representativity, a limitation that could be overcome by aerial and satellite systems [2].

In the past, the requirements for remote sensing data to support precision agricultural management were defined as a temporal resolution of not more than two weeks [3] and a spatial resolution of not coarser than 20 × 20 m^2^ [4], ideally about 10 × 10 m^2^ [3]. With a resolution between 10 × 10 m^2^ and 20 × 20 m^2^ in the relevant spectral bands and a revisit interval of 2–3 days, one would assume that these conditions are fulfilled by the Sentinel-2 satellites [5]. Another promising, recently advancing remote sensing technology are unmanned aerial vehicles (UAVs), being able to provide data with high-resolution at constantly decreasing acquisition costs [6]. Yet, both systems are subject to certain limitations: satellite-based data acquisition depends on cloud coverage, while the UAV’s application is limited to relatively small areas and available resources.

One of the most frequently considered canopy characteristics by remote sensing approaches is the green area index (GAI), being a key parameter for modeling yield-relevant processes, such as radiation interception, evapotranspiration and consequential growth [7,8]. Therefore, it is possible to predict crop yield by the combination of interpolated GAI-values with weather data [9].

GAI can be assessed by multispectral measurements, whether by simple linear regression between GAI and different vegetation indices (VI) or by multivariate methods [10,11,12]. Even though different effects impeding GAI-calibration are discussed in the literature, e.g., [13,14,15,16], quite stable GAI-calibrations for cultivars of the same crop over the whole season are achievable [17,18]. However, the parameters of the empirical model equations may vary with crop, background effects, spatial resolution and VI [19,20,21].

The calibration of satellite data is difficult as it requires the upscaling of point measurements to the same scale as the satellite measurements. Hence, multiple point measurements have to be carried out to represent one satellite pixel. This results in a high workload and yet relatively small datasets. Some studies try to bypass this by using radiative transfer models to simulate reflectance values as one would expect them to be measured by satellites to correlate them subsequently with ground measurements [22,23]. However, a simulation model cannot depict potential problems arising from the remote sensing method, such as atmospheric effects [23,24].

UAV-based spectral reflection measurements might offer an opportunity to close this data gap, being able to provide a larger number of area-representative values in a short time with a comparably small workload. Therefore, the possibility of comparing satellite with UAV data is of growing interest [25,26]. Revill et al. [27] introduced in 2020 a two-step procedure for the calibration of satellite VIs to crop canopy characteristics: calibrate UAV data with ground measurements and afterwards using the UAV predictions to calibrate the satellite data. Such a new methodological framework might give new insights into the possible contribution of the Sentinel-2 data with regard to crop monitoring. Further refined, it should additionally enable the contextualization and comparison of UAV- and satellite-based spectral data within the application of yield prediction. The main starting points of this study are to transfer the approach of Revill et al. [27] to a larger data volume, including multiple years and many acquisition dates, and to make use of well-calibrated UAV-based multispectral VI algorithms [16].

Therefore, the objectives of this study were: (I) to demonstrate the possibility to calibrate empirical VI models for GAI-estimation from Sentinel-2 data by the combined use of UAV data and a corresponding validated prediction model, (II) to evaluate the GAI-model based on a large data volume, enabling the identification of problems with regard to the GAI-models’ applicability to different satellite acquisition dates and over the whole season, (III) to assess the spatial and temporal resolution of Sentinel-2 data for GAI monitoring, and (IV) to estimate the potential and limitations of satellite-based GAI-data for yield prediction in the context of precision agriculture.

## 2. Materials and Methods

### 2.1. Data Acquisition

#### 2.1.1. Study Sites

**Figure A1:**
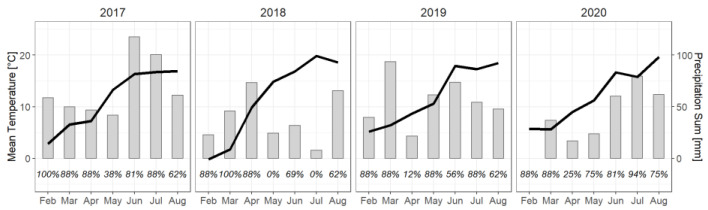
The weather during data acquisition at the DWD-station Kiel-Holtenau [56]. Bars indicate the monthly precipitation sum, lines the mean monthly temperature 2 m above ground, and the percent values indicated at the bottom the monthly median of cloud coverage between 10–11 a.m. (hour of Sentinel-2 overflight), measured in one eighth of the monitored sky.

Data acquisition was conducted from the first of February until harvest during four seasons (2016–2020) at the Hohenschulen Experimental Farm of the Kiel University (10.0 E, 54.3 N, 30 m a.s.l.). The study site is located in Northern Germany ([28]: long-term annual average: temperature = 8.9 °C, precipitation = 788 mm). The weather conditions differed distinctly between the years, 2017 having many cloudy days and high amounts of precipitation and with a particularly cloudless, dry and warm summer in 2018 (in Appendix A, Figure A1).

For the purpose of calibrating and evaluating a GAI-model for Sentinel-2 data, the UAV data were taken from adjacent areas of plot trials from the Hohenschulen Experimental Farm. These area sections are managed uniformly according to regional farmer’s practice. They are of varying extent and mostly small (18 different area sections with 0.04–5.71 ha, Table A1 and Table A2), and the number of UAV-based acquisition dates per area section differs (1–10 dates).

For further analysis, data of the most frequently UAV-based assessed plot trial were used. The trial tests the interaction of winter wheat genotypes (2017: 220 genotypes, 2018 and 2019: 52 genotypes) with different management strategies (2017: three differing management intensities, 2018 and 2019: four management intensities). These trials are characterized in more detail by Rose and Kage [18]. Due to this wide range of genotype-management combinations, each three times replicated, the trials covered a large area, even in the context of satellite-based data acquisition. Contextualizing the trials’ spatial extent by the 20 × 20 m^2^-grid of the Sentinel-2 Red Edge (RE)-pixels this means: in 2017, 69 Sentinel-2 RE-pixels were completely within the area of the field trial, in 2018 13 RE-pixels and in 2019 20 RE-pixels. All available UAV and satellite data between first of February and harvest date were collected for these trial sites (Table A3). In each of the three seasons, a large number of UAV overflights were performed, but since only two satellite dates were available in 2017, further analysis was restricted to the data of 2018 and 2019.

#### 2.1.2. UAV-Based Multispectral Data

A fixed wing UAV (eBee from senseFly, Lausanne, Switzerland) was applied as carrier system for a multispectral camera (Parrot Sequoia, Parrot Drones SAS, Paris, France). The four measured wavebands are green (550 nm, bandwidth: 40 nm), red (660 nm, bandwidth: 40 nm), RE (735 nm, bandwidth: 10 nm) and near-infrared (NIR, 790 nm, bandwidth: 40 nm). The spatial resolution was 8 × 8 cm^2^. The Sequoia camera has an integrated sunshine sensor and thus provides reflection values as fraction of the incoming radiation. For calibration purposes, images of a grayscale target were made before every flight. The flight height and course were adjusted to acquire at least five images per ground position. A post flight-manager (eMotion3, SenseFly SA, Switzerland) was used to assign the location and orientation of the UAV to each image and to include RINEX-files (Receiver Independent Exchange Format) to obtain high-accuracy georeferenced data (geolocation uncertainty < 5 cm with 1-sigma). Reflectance maps of the four different wavebands were generated using the photogrammetry software Pix4Dmapper (Pix4D SA, Switzerland).

#### 2.1.3. Satellite-Based Multispectral Data

The Sentinel-2 mission is composed of two identical satellites launched by the European Space Agency (ESA) in June 2015 and March 2017. Since 2017, they provide multispectral data of thirteen different bands in a return interval of 2–3 days in midlatitudes [5]. The satellite data used in this study were downloaded free of charge as an L2A-product, hence already processed for atmospheric correction. Due to their high resolution and their relevance for the GAI-prediction [3,22,29], the selected bands were green (B3: 560 nm, bandwidth: 36 nm, resolution: 10 × 10 m^2^), red (B4: 665 nm, bandwidth: 31 nm, resolution: 10 × 10 m^2^), RE (B6: 740 nm, bandwidth: 15 nm, resolution: 20 × 20 m^2^) and NIR (B8: 842 nm, bandwidth: 106 nm, resolution: 10 × 10 m^2^). Satellite data were only used for further analysis if the whole experimental station could be considered as free of clouds, cloud shadows, snow and ice (using the scene classification map of the Sentinel Hub EO-browser, a result of the ESA scene classification algorithm).

**Figure 1:**
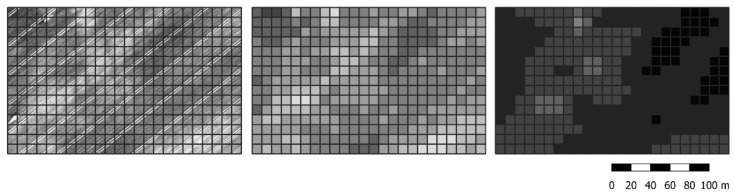
Section of a near-infrared (NIR) map and the generated grid-shapefile (white: 0.4–black: 0.85), Left: unmanned aerial vehicle (UAV)-provided resolution, Centre: UAV data averaged to 10 × 10 m^2^ grid, Right: Sentinel data.

To combine satellite and UAV data, grid-shapefiles for each covered area were created in QGIS, version 3.8.0 [30], fitting to the 10 × 10 m^2^- and 20 × 20 m^2^-grid of the Sentinel-2 data (Figure 1). The UAV data of the same grid element were band- and date-wise averaged. Likewise, all Sentinel-2 reflection maps were transferred in the grid of both resolutions. The 10 × 10 m^2^-grid shapefile was even applied to the satellite-based RE-maps, as when combined with the other three bands, GAI-predictions should vary at a resolution better than 10 × 10 m^2^. Problematic grid elements were identified based on the UAV data (including field margins and areas with shadows or weeds) and excluded from further considerations.

#### 2.1.4. Ground Truth Data

The GAI-calibrations applied to the UAV data are based on extensive destructive sampling in 11 different cultivars, 7 nitrogen levels and 6 sowing densities, described in more detail by Bukowiecki et al. [16]. During three years, the manually determined GAI on the 0.25 m^2^-sampling spots was correlated with UAV-based spectral data with a spatial resolution within the centimeter range. For the calibration of UAV-spectral data to GAI, 474 samples originating from 13 different sampling dates were used.

**Figure 2:**
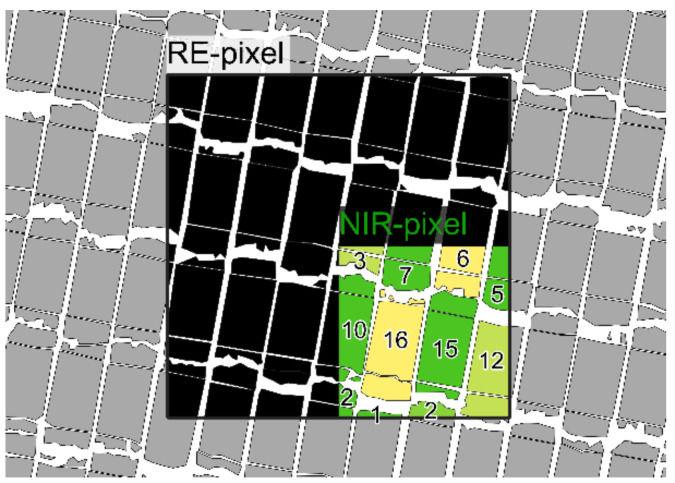
Section of a plot-shapefile with an exemplarily indicated NIR- and Red Edge (RE)-grid element. For the NIR-pixel, the percentage area shares of the different plots for the generation of the yield map are given.

Yield data of the closer considered plot trials were used for further analysis: to upscale the yield to the same grid as the multispectral data, detailed shapefiles of the trial structure were created, based on the UAV flight with the highest mean GAI of the season (Figure 2). Therewith, the area proportion of bare soil and different plot segments of each grid element were determined and then multiplied with its respective grain yield, assigning a value of zero to bare soil segments.

### 2.2. Statistical Analysis

Data analysis was done completely in R, version 3.6.1 [31]. All statistical tests were performed with a level of significance of *p* = 0.05. The statistical measures used are the mean absolute error (*MAE*, Equation (1)) and the coefficient of determination (R^2^, Equation (2)). The *MAE* quantifies the deviation between measured values (*x_i_*) and predictions (*y_i_*), and R^2^ indicates how much variation of the measured values is explained.

(1) $$MAE=\overset{n}{\underset{i=1}{\sum }}\frac{\left|y_{i}-x_{i}\right|}{n}$$

(2) $$R^{2}=\frac{\sum_{i=1}^{n}{\left(y_{i}-\bar{y}\right)}^{2}}{\sum_{i=1}^{n}{\left(x_{i}-\bar{y}\right)}^{2}}$$

where *n* is the number of observations included in the calculation and $\bar{y}$ is the average of the predictions.

**Figure 3:**
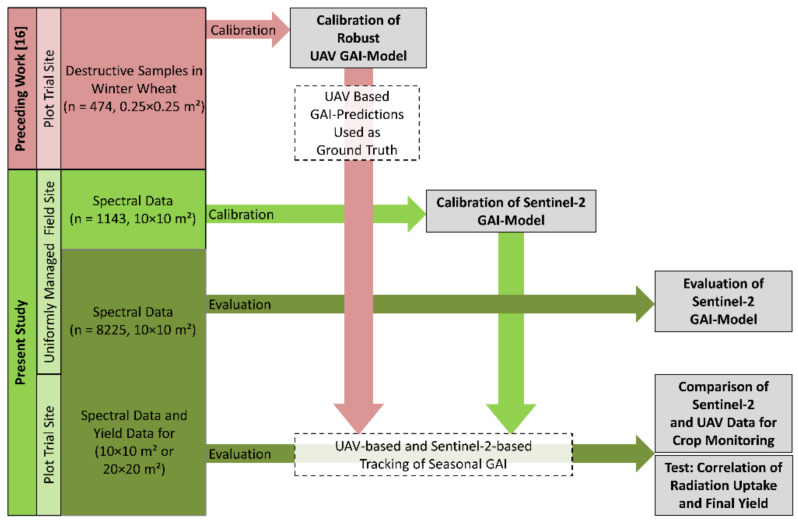
Overview of datasets and workflow (red: precedent work by Bukowiecki et al. [16], green: present study). The different objectives are depicted by grey boxes.

The UAV-based GAI was calculated using the empirical VI models of Bukowiecki et al. [16] and further used as reference data for the satellite-derived estimates. For calibration and evaluation purposes, UAV and satellite data were assigned to each other if their acquisition dates differed not more than five days as the plant development during this period can be neglected. An overview of the different steps of the analysis and datasets used is given in Figure 3.

#### 2.2.1. GAI-Model for Sentinel-2 Multispectral Data

The UAV and Sentinel-2 data were divided in calibration and evaluation datasets. All observation dates with UAV data destructively evaluated by Bukowiecki et al. [16] were used as calibration data (six satellite dates and 1143 elements, Table A1). All other data (23 satellite dates and 8225 elements, Table A2) were used for evaluation. Different approaches were tested to calibrate satellite data to the UAV-based GAI (Table 1): linear models based on one Simple Ratio (SR), one linear model based on three SRs ([16]: VIQUO), and three models with an exponential term ([11]: RENDVI, [32]: NDVI, [33]: EVI2), taking into account the known non-linear behavior of the these VI-approaches (e.g., [11]). EVI2 was selected due to its reputation to be robust to atmospheric influences [33]. The different models were compared by their MAEs in terms of calibration and evaluation and the best performing model was selected for further analysis.

To test for significant effects of the acquisition date in the evaluation dataset, a linear model was set up for each VI, with GAI as the response variable and the VI and the acquisition data as predictors (without interaction). Using the package lsmeans [34], an ANOVA was applied to these linear models and Tukey-adjusted comparisons were used to conduct a multiple comparison of the interaction between the VI and dates (slopes).

#### 2.2.2. Application of Satellite and UAV Data for Crop Monitoring

To get an insight into the comparative potential contribution of UAV and satellite data to GAI-monitoring and yield forecasting, we evaluated the possibility to predict grain yield from the total absorbed effective photosynthetic radiation, derived from UAV- or satellite-based GAI-estimates. To identify possible reasons for deviations between satellite and UAV-based results, three spatial, two temporal and two spectral approaches and their combinations were tested (Table 2): following the spatial requirements for precision farming proposed in the literature [3,4], we tested the UAV and satellite data to explain the yield at the resolution of 10 × 10 m^2^ and 20 × 20 m^2^. Hence, UAV and satellite data were averaged in the 20 × 20 m^2^-grid of the Sentinel-2 RE-band or in the 10 × 10 m^2^-grid of its other bands (Figure 1). The GAI was calculated with the NIR/RE- or VIQUO-models for the Sentinel-2 data (section “GAI-Model for Sentinel-2 Multispectral Data”), respectively, with the GAI-models for UAV data of Bukowiecki et al. [16]. If a GAI < 0 m^2^/m^2^ was predicted for a grid element, the value was set to 0 m^2^/m^2^. In the next step, different GAI-data compilations were generated, combining the different spectral-, spatial- and temporal options (Table 2). The datasets were complemented by assuming a GAI of 0 m^2^/m^2^ at harvest and a GAI of 0.3 m^2^/m^2^ at the first of February, respectively, the GAI of the first acquisition date if <0.3 m^2^/m^2^. Hourly data of a local weather station were used to calculate the thermal time (base temperature = 0 °C) and the weighted incoming photosynthetic active radiation to account for the temperature dependency of photosynthesis ([35]: a trapezoidal weighting function of the daily mean temperature ranging from 0 to 1 was used, with transition points at 2.5, 9.5, 20 and 35 °C).

Then, the GAI was interpolated linearly for each remote approach, and the absorbed effective photosynthetic radiation was calculated according to Lambert–Beer’s law (assumed extinction coefficient = 0.7) and cumulated in daily time steps until harvest. The proportion of the grain yield variance explained by the calculated radiation uptake was estimated by linear models.

## 3. Results

### 3.1. Sentinel-2 GAI-Predictions: Overall Calibration

On average, the error of the different VI-based GAI-predictions in calibration and evaluation are mostly below 1 m^2^/m^2^ (Table 3). During calibration, the best performing models are the exponential models based on the NIR- and Red-band. However, the NDVI and the EVI2 are both outperformed by the NIR/RE- and the VIQUO-based linear models in terms of evaluation. Comparing these two VI-models (Table 3), both approaches provide the same MAEs in terms of calibration and evaluation (MAE_calibration_ = 0.52 m^2^/m^2^, MAE_evaluation_ = 0.52 m^2^/m^2^). Consequently, the simpler NIR/RE-model is considered as the most efficient performing model and is selected for further examinations.

**Figure 4:**
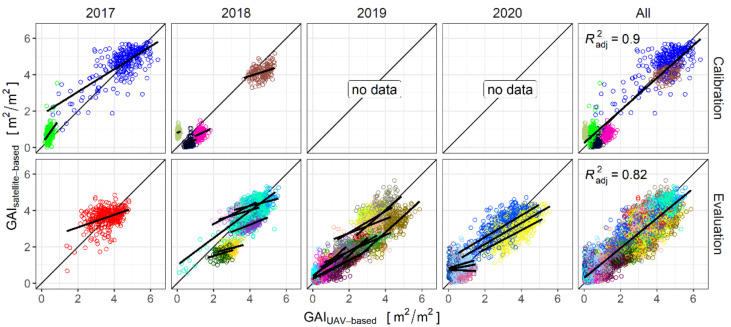
Correlation between UAV- and satellite-based (NIR/RE) predicted GAI for calibration and evaluation, split by years and for the whole datasets. Colors indicate different Sentinel-2 dates. For the single years, the linear regression lines of the different acquisition dates are given, in the comprehensive plot the joint linear regression and the coefficient of determination are indicated.

The NIR/RE-model reaches a high correlation between UAV-based and satellite-based GAI-predictions (R^2^_calibration_ = 0.9, R^2^_evaluation_ = 0.82) (Figure 4). The calibrated and evaluated regression model outputs were consistent, indicating a stability of this general GAI-model. However, the results of the single acquisition dates scatter considerably around the 1:1-line. Furthermore, with regard to the date-specific GAI-variation, the satellite data provide rather scattered point clouds, shifted or rotated in relation to the 1:1-line (Figure 4). The single-date R^2^-values vary between 0.01–0.62 in the calibration and between 0.01–0.55 in the evaluation dataset.

The ANOVA revealed a significant effect of the acquisition date and the interaction between VI and date (*p* < 2.2e^−16^) for all tested VIs. The Tukey-adjusted comparisons classified the 23 satellite dates in at least eleven different groups, suggesting that date-specific effects appear frequently.

### 3.2. Application of Satellite and UAV Data for Crop Monitoring

Cloudy conditions limited the applicability of Sentinel-2 data for crop monitoring during 2017 (Figure A1, Table A3). In 2018 and 2019, it was possible to obtain relatively well temporal-resolved UAV-based and satellite-based GAI-courses (Table A3). While these courses display relatively similar average values, some general differences exist: the UAV-based GAI-courses reach their maximal value earlier, while the satellite-based GAI is higher in the late season. These differences in the GAI-courses lead to differences in the calculated total absorbed effective photosynthetic radiation and to varying explanatory contributions with regard to the final grain yield variation. The yield ranged on the 10 × 10 m^2^-grid between 391–616 g/m^2^ in 2018 and 397–772 g/m^2^ in 2019. On the 20 × 20 m^2^-grid the variation was considerably lower (2018: 453–571 g/m^2^, 2019: 458–666 g/m^2^).

**Figure 5:**
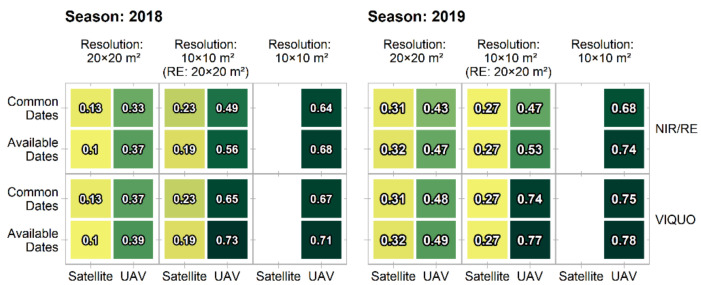
R^2^-values of the linear regressions between the total absorbed effective photosynthetic radiation and the final grain yield. Differences arise upon the modified UAV and satellite data basis (Table 2).

UAV- and Sentinel-2 based calculated radiation uptakes display in 2019 higher correlations with the grain yield than in 2018 (Figure 5). Due to year- and site-effects, such differences in the yield-radiation uptake correlation are likely to appear. However, the comparison of the results of the UAV- and Sentinel-2 based calculations reveals similar patterns in both years (Figure 5): the radiation uptake-yield-correlation for each tested option is higher when calculated with the UAV-based approach (R^2^_2018_ = 0.33–0.73, R^2^_2019_ = 0.43–0.78) compared with the satellite-based approach (R^2^_2018_ = 0.1–0.23, R^2^_2019_ = 0.27–0.32) and even the UAV-based approach based on the most reduced data volume explains still more than the best performing satellite-based approach. For both remote sensing approaches, the variation of the spatial resolution has a larger effect than the spectral or temporal aspect. The UAV-based R^2^ values display the gradient one would expect - decreasingly explained yield variation when the spatial or temporal resolution of the data basis is reduced. The satellite-based approach reacts less straightforwardly: while in 2018, the lower spatial resolution results in less explained yield variation, the option has the opposite effect in 2019. Further, the reduction of the considered acquisition dates decreases the R^2^-values in 2019 but increases them in 2018. Additionally, there are no distinct differences between the different spectral options in both years (only at the third decimal position).

## 4. Discussion

### 4.1. Concept of UAV-based Calibration and Evaluation of Satellite Data

The spatial and temporal resolution of spectral data is of high importance for their application in crop management. UAV-based data acquisition, in principle, can provide data at any desired spatial and temporal resolution—leaving aside workload and weather conditions. Satellites, here the Sentinel-2 data, have a rigid and in most cases coarser resolution. This affects both, calibration of prediction models and application in crop management. In Section 3.1 it was demonstrated that by the two-step approach proposed by Revill et al. [27], refined by a UAV-GAI-calibration [16], large datasets can be created, enabling further investigations about the information content of satellite data (as for example in Section 3.2).

### 4.2. Accuracy of the GAI-Prediction

Our results indicate that a suitable model for satellite-based GAI-prediction has to include the RE-band despite its coarser spatial resolution of 20 × 20 m^2^, compared to the 10 × 10 m^2^ resolution of the visible wavebands. VIs such as the NDVI and the EVI2 were outperformed by the simple NIR/RE-ratio (Table 3). This general finding is consistent with several studies [36,37]. The added value by the RE-bands can be traced back to its characteristic to penetrate deeper into canopies, hence to saturate less at high crop densities [10,38]. The prediction error of 0.52 m^2^/m^2^ is slightly better than that of previous studies with winter wheat (e.g., [12]) and other crops [39,40,41] and matches well to the results of Revill et al. [27]. In contrast to the findings of Richter et al. [22] and Verrelst et al. [39], introducing additional bands did not enhance the GAI-prediction (Table 3). This could be examined further, considering the full spectrum of Sentinel-2 bands and calibration methods—for example, machine learning regression algorithms [12,42]. However, other studies already demonstrated good results applying simple VI-approaches to Sentinel-2 data [11,22] and it should be kept in mind that a simple but still relative well working VI-approach is easier to communicate and has the advantage of lower download- and processing time [39].

Yet, the analysis revealed several deficiencies of the NIR/RE-model, raising the topics of temporal and spatial resolution. Apparently, the frequent statement that the GAI-course over the season can be well mapped with Sentinel-2 data [3,11,12,21,29,37,40,41,42,43,44,45] is based on large differences and continuously increasing GAI-values between different sampling dates. Yet, the Sentinel-2 based single-date GAI-estimations are at some dates systematically biased and the true GAI-variation is in most cases considerably underestimated (Figure 4). This effect also appears in the calibration dataset, whose UAV-based GAI-predictions were destructively evaluated [16] and are therefore a reliable reference. Date-specific clustered patterns were also detected by Revill et al. [27] and by Dimitrov et al. [37]. Other studies describe date-specific varying precision in GAI-prediction, e.g., [43]. This is no general problem of multispectral measurements, as many studies already presented approaches to predict GAI across a wide range of growth stages with one single calibration model [3,15,16,20]. Campos-Taberner at al. [40] traced a sudden GAI-drop at one satellite acquisition date back to a suboptimal atmospheric correction of the Sentinel-2 data or non-identified clouds. However, in our dataset the problem arose not only on one, but on a high number of acquisition dates and was not correctable by the choice of the VI, not even by the EVI2 that has the reputation to be robust to atmospheric influences [19,33].

This raises the question why so far, as to say based on the literature research carried out [3,11,12,21,29,37,40,41,42,43,44,45], the impact of date-specific effects when working with Sentinel-2 data is not further discussed. The common practice of distributing the sampling dates over different BBCH stages (e.g., [11,29]) and combining data of different locations, years and cultivars [40,41,43,44,45] might have disguised this problem so far—thereby, even if a certain shift between different satellite data acquisitions is detected, it cannot be clearly distinguished from other reasons, such as a starting VI-saturation at high GAIs, different VI-GAI-correlations at differing BBCH stages, or altering soil background. Furthermore, the significant effects of measurement date to the relationship VI-GAI can be concealed by a low number of samplings per date (the lower the number of samplings per date, the more unlikely is the rejection of the null hypothesis of equality of slope and intercept).

### 4.3. Spatial and Temporal Resolution of Sentinel-2 GAI-Predictions

#### 4.3.1. Spatial Resolution

The finding that the spatial variation at single days is not well met by the Sentinel-2 data matches the results of Delloye et al. [46]. Furthermore, they depict the same problem by the introduction of the RE-band in the GAI-prediction: a significant informational value added in the description of the GAI-development through the season, but at the same time a considerable reduction of accuracy in describing the spatial heterogeneity.

Additionally, inaccurate georeferencing might affect the precision of local GAI predictions. Gascon et al. [47], expect a geolocation uncertainty of the Sentinel-2 data in most cases between 4.5–8 m (2-sigma), Pandžic et al. [48] describe deviations between 6–12.7 m. Such spatial shifts may already alone put into question the potential of the Sentinel-2 data to achieve an adequate spatial resolution for precision farming. This problem could be minimized via further georeferencing steps, but we restricted our analysis to the data quality made available for public use.

#### 4.3.2. Temporal Resolution

Due to cloud coverage a time interval of two weeks was rarely achieved and several times, periods of more than one month exist without usable data. This difficulty does not only concern Northern Germany; for example, Clevers et al. [13] in the Netherlands and Hunt et al. [49] in UK faced the same problem when working with Sentinel-2 data. The importance of those gaps depends not only on their duration, but of the timing: not each acquisition date might have the same informative value for yield prediction [50,51]. In 2017, the generation of a GAI-course was not possible due to this limiting factor, highlighting the restraining power of the temporal resolution in the context of GAI-monitoring. Besides this aspect, the frequent occurrence of date-specific effects in the satellite data calls into question the usability of the available data.

### 4.4. Application of Satellite and UAV Data for Crop Monitoring

The finding that the Sentinel-2 data meet neither the spatial, nor the temporal resolution required by the literature for precision agriculture [3,4] raises the key question of the contribution of Sentinel-2 data to crop monitoring and yield prediction. So far, different studies have considered the Sentinel-2 data as valuable input for yield estimation on a landscape and field level [49,51,52]. However, each of these analyses was to a certain extent limited; basing upon data of only one satellite acquisition date or only one season or not considering the RE-band.

Relating the yield with the radiation uptake is a retrospective approach, hence is not applicable to forecast the yield in independent seasons. Still, it is a possibility to get an insight to the possible contribution of a multispectral crop monitoring for yield prediction. However, yield maps from combined harvesters are known for errors (e.g., [51,53,54]) and so far, there is no common correction procedure [49]. In order to reference remote sensing data with reliable and accurate yield data, we introduced an alternative method of generating yield maps by plot yields and UAV-based crop cover maps. The absolute performance of spectral data might not be adequately assessed by this approach—as the variation of the GAI in the plot trials (bare soil, cultivar, fertilization) clearly exceeds the variation in uniformly managed fields. Nevertheless, as the data of both remote sensing platforms are equally compared to these yield maps, the relative comparison of both methods is valid.

By calculating the total absorbed effective photosynthetic radiation for the same trial sites, it could be shown that in both years the UAV-based spectral data explains a larger share of the grain yield variation—in most scenarios one third to one half more than the respective Sentinel-2-based approach. Escolà et al. [52] traced low correlations between Sentinel-2 data and yield data back to the problem of VI-saturation. Yet, their proposal of using RE-wavebands did not lead to a better performance in our analysis. However, the distinct reduction of yield variation UAV-based explained when upscaling its RE-band to the same 20 × 20 m^2^-resolution as the one of the Sentinel-2 points out the central importance of this band.

In the literature, fewer spectral information is partly traced back to broader spectral bands [17,55]. This effect cannot be clearly assessed as the Sentinel-2 data in the green-, red- and RE-bands are slightly narrower than those of the UAV-based multispectral camera, meanwhile its NIR-band is much broader. The narrower Sentinel-2 NIR-bands have the drawback of a lower spatial resolution.

Finally, the increasing performance of the satellite data with reduced acquisition dates in 2018 indicates an additional need to analyze further the impact of date-specific affected dates. A possible solution is to use a concurrently performed UAV flight to identify and recalibrate date-specific shifted Sentinel-2 data or to enhance the spatial resolution as proposed by Khaliq et al. [26]. If it is achievable to compensate the difficulties of Sentinel-2-based GAI-prediction entirely is an open question which can only be answered on the basis of large evaluation datasets. Until then, a cautious use of Sentinel-2 data in the context of precision agriculture is advisable. Hence, despite obvious advantages of satellite-based data acquisition, UAV-driven approaches should be preferred at the present time and at the current state of knowledge.

## 5. Conclusions

Crop management decisions, especially on the scale of precision agriculture, require reliable, accurate crop data with an adequate spatial and temporal resolution. The presented UAV-based approach enables an assessment of the Sentinel-2 spectral data with regard to these requirements.

On the basis of a large quadrennial dataset, a model to derive the GAI of winter wheat from Sentinel-2 data was calibrated and evaluated. A simple ratio approach based on the NIR- and RE-bands performed best, enabling the calculation of stable GAI-courses through the season. However, the spatial variation is not properly depicted within different dates. Further analysis revealed that the Sentinel-2 data fall short of expectations for precision farming. By a comparative consideration of UAV data, it could be shown that especially the coarse resolution of the RE-band limits the possible contribution of Sentinel-2-based crop monitoring to yield prediction. Due to cloud coverage, also the temporal resolution of the Sentinel-2 data is frequently poor. Excluding date-specific affected satellite data would further decrease the provided time interval. Until a framework for the handling of these issues is set out, a careful use of the Sentinel-2 data is advisable.

## Figures and Tables

**Table 1:** Tested vegetation indices to calibrate Sentinel-2 data to the UAV-based GAI of winter wheat (obtained by the combination of UAV data and a corresponding GAI-calibration [16]).

Vegetation Index	GAI-Model
NIR/Green	GAI = a + b × NIR/Green
NIR/Red	GAI = a + b × NIR/Red
NIR/RE	GAI = a + b × NIR/RE
NDVI	GAI = a + e ^((NIR - Red)/(NIR + Red))^
EVI2	GAI = a + e^(b × 2.5 × (NIR - Red)/(NIR + 2.4 × Red + 1))^
RENDVI	GAI = a + e^(b × (NIR - RE)/(NIR + RE))^
VIQUO	GAI = a + b × NIR/Green + c × NIR/Red + d × NIR/RE

**Table 2:** Tested concepts to modify the UAV and satellite data base. Each of the options of one aspect can be combined with every option of the other aspects, resulting in 8 different satellite and 12 UAV scenarios. All acquisition dates and the selected dates for the “Common Dates”-option are listed in Table A3.

Aspect	Tested Options	Abbreviation
Temporal	All available UAV and satellite acquisition dates	Available Dates
	Only UAV and satellite acquisitions dates differing not more than 5 days	Common Dates
Spatial	Resolution of yield map and spectral data: 10 × 10 m^2^	Resolution: 10 × 10 m^2^
	UAV and satellite RE-band: 20 × 20 m^2^,	
	all data: 10 × 10 m^2^	Resolution: 10 × 10 m^2^
Spectral	(RE: 20 × 20 m^2^)	
	Resolution of yield map and spectral data: 20 × 20 m^2^	Resolution: 20 × 20 m^2^

**Table 3:** Models for GAI-prediction with Sentinel-2 data and their Mean Absolute Errors (MAE) in calibration and evaluation.

Vegetation Index	MAE_calibration_ [m^2^/m^2^]	MAE_evaluation_ [m^2^/m^2^]	GAI-Model
NIR/Green	0.73	1.04	0.2040 + 0.2179 × NIR/Green
NIR/Red	1.13	1.26	1.13776 + 0.0639 × NIR/Red
NIR/RE	0.52	0.52	−9.781 + 8.712 × NIR/RE
NDVI	0.38	0.56	0.09 × e^(4.1858 × NDVI)^
RENDVI	0.66	0.72	0.2908 × e^(10.9942 × RENDVI)^
EVI2	0.43	0.98	0.2638 × e^(2.4013 × EVI2)^
VIQUO	0.52	0.52	−9.236087 − 0.023062 × NIR/Green − 0.002741 × NIR/Red + 8.142750 × NIR/RE

## Data Availability

The code used and the datasets generated during the different steps of the analysis are available from the corresponding author on reasonable request.

## Appendix A

**Table A1:** Acquisition dates and data volume for the calibration of the GAI-models.

Sentinel-2 Date	UAV Date	Number of Area Sections (Number of Grid Elements, Size = 10 × 10 m^2^)
2017-06-02	2017-05-29	4 (274)
2017-07-19	2017-07-17	4 (277)
2018-04-18	2018-04-16	1 (148)
2018-04-20	2018-04-23	1 (148)
2018-05-23	2018-05-22	1 (148)
2018-07-17	2018-07-17	1 (148)
Total Number of Grid Elements (Area)		1143 (11.43 ha)

**Table A2:** Acquisition dates and data volume for the evaluation of the GAI-models.

Sentinel-2 Date	UAV Date	Number of Area Sections (Number of Grid Elements, Size = 10 × 10 m^2^)
2017-06-02	2017-06-02	1 (345)
2018-05-05	2018-05-02	2 (130)
2018-05-05	2018-05-03	1 (148)
2018-05-20	2018-05-16	1 (148)
2018-05-23	2018-05-24	4 (733)
2018-06-02	2018-06-01	1 (148)
2018-06-07	2018-06-06	2 (130)
2018-06-07	2018-06-12	1 (148)
2019-02-27	2019-03-01	2 (223)
2019-03-31	2019-04-01	2 (137)
2019-03-31	2019-04-02	6 (251)
2019-04-08	2019-04-08	3 (171)
2019-04-08	2019-04-10	5 (162)
2019-04-18	2019-04-16	5 (369)
2019-04-23	2019-04-25	5 (200)
2019-05-15	2019-05-15	4 (175)
2019-05-18	2019-05-23	4 (189)
2019-06-02	2019-05-29	6 (293)
2019-06-14	2019-06-13	4 (300)
2019-06-29	2019-06-25	3 (88)
2019-07-24	2019-07-23	4 (237)
2020-03-23	2020-03-23	3 (379)
2020-04-07	2020-04-06	3 (335)
2020-04-17	2020-04-17	2 (687)
2020-05-29	2020-05-29	2 (541)
2020-06-01	2020-06-02	3 (653)
2020-06-23	2020-06-23	2 (905)
Total Number of Grid Elements (Area)		8225 (82.25 ha)

**Table A3:** Sentinel-2 and UAV data acquisition dates of the trial sites. Dates of the same month and year are opposed to emphasize the achieved temporal resolution of both remote sensing approaches.

Year	Month	Sentinel-2 Date	UAV Date
2017	April	-	04, 19
	May	-	07, 15, 23, 29
	June	02	14, 19, 20, 27
	July	19	03,13, 17, 21, 28
2018	March	-	09
	April	08 *, 18 *, 20 *	09 *, 16 *, 23 *
	May	05, 08, 13, 15 *, 20, 23 *, 25, 30	03, 16 *, 22 *
	June	02 *, 07 *	01* , 06 *, 12, 20, 26
	July	17 *, 24	05, 13, 17 *
2019	February	27 *	18
	March	31*	01*, 19
	April	08 *, 18 *, 23, 25 *	02 *, 08 *, 16 *, 25 *
	May	15 *, 18	03, 13, 15 *, 23, 29
	June	02 *, 14 *, 29 *	05 *, 13 *, 21, 26 *
	July	24 *	03, 09, 17, 23 *

* Dates selected for the “Common Dates”-analysis.
